# Measurement and Correction of Stooped Posture during Gait Using Wearable Sensors in Patients with Parkinsonism: A Preliminary Study

**DOI:** 10.3390/s21072379

**Published:** 2021-03-30

**Authors:** Se Hoon Kim, Seo Jung Yun, Quoc Khanh Dang, Youngjoon Chee, Sun Gun Chung, Byung-Mo Oh, Keewon Kim, Han Gil Seo

**Affiliations:** 1 Department of Rehabilitation Medicine, Seoul National University Hospital, Seoul 03080, Korea; whyamihappy@naver.com (S.H.K.); sjselena0611@gmail.com (S.J.Y.); suncg@snu.ac.kr (S.G.C.); keepwiz@gmail.com (B.-M.O.); keewonkimm.d@gmail.com (K.K.); 2 MKS Instruments Korea, 543 Beonji, Daedeok Techno Valley, Yongsan-dong, Yuseong-gu, Daejeon 34028, Korea; khanhdq8689@gmail.com; 3 School of Electrical Engineering, Biomedical Engineering, College of Engineering, University of Ulsan, Ulsan 44610, Korea; yjchee@ulsan.ac.kr; 4 National Traffic Injury Rehabilitation Hospital, Yangpyeong-gun 12564, Korea

**Keywords:** posture, neurologic disorders, biomechanics, gait/balance

## Abstract

Stooped posture, which is usually aggravated during walking, is one of the typical postural deformities in patients with parkinsonism. However, the degree of stooped posture is difficult to quantitatively measure during walking. Furthermore, continuous feedback on posture is also difficult to provide. The purpose of this study is to measure the degree of stooped posture during gait and to investigate whether vibration feedback from sensor modules can improve a patient’s posture. Parkinsonian patients with stooped posture were recruited for this study. Two wearable sensors with three-axis accelerometers were attached, one at the upper neck and the other just below the C7 spinous process of the patients. After being calibrated in the most upright posture, the sensors continuously recorded the sagittal angles at 20 Hz and averaged the data at every second during a 6 min walk test. In the control session, the patients walked with the sensors as usual. In the vibration session, sensory feedback was provided through vibrations from the neck sensor module when the sagittal angle exceeded a programmable threshold value. Data were collected and analyzed successfully in a total of 10 patients. The neck flexion and back flexion were slightly aggravated during gait, although the average change was <10° in most patients in both measurement sessions. Therefore, it was difficult to evaluate the effect of sensory feedback through vibration. However, some patients showed immediate response to the feedback and corrected their posture during gait. In conclusion, this preliminary study suggests that stooped posture could be quantitatively measured during gait by using wearable sensors in patients with parkinsonism. Sensory feedback through vibration from sensor modules may help in correcting posture during gait in selected patients.

## 1. Introduction

Postural deformities are one of the major abnormalities observed in >30% of patients with Parkinson’s disease (PD) and may result in stooped posture, dropped head syndrome and Pisa syndrome [1,2]. The prevalence of camptocormia, an abnormal flexion of the thoracolumbar spine during standing and walking, is known to be around 3–18% in patients with PD [3]. Unlike kyphoscoliosis, stooped posture is mostly reversible and can be corrected if patients are directed to stand upright or lean against the wall. However, the posture usually worsens again as the time spent standing or walking increases.

The possible pathogenesis of stooped posture in PD can involve a mechanism of the central nervous system such as overactivity of the flexor muscles due to abnormal activity of the basal ganglia, a form of dystonia, paraspinal myopathy and medications in PD [3,4]. There are many views as to why stooped postures occur in patients with PD. Some argue that the stooped posture is a reward strategy to improve gait initiation and balance [5]. However, it is known that a stooped posture can cause back pain (one of the most common types of pain in PD) [6,7], gait disturbance, dysphagia and even dyspnea [1,8]. Although many treatments have been proposed to date for other symptoms such as gait and balance problems [9], there is still no clearly proven treatment for stooped posture in patients with parkinsonism. Moreover, there has been no consistent method for evaluating the effect of treatment for stooped posture [3]. Even in recent studies, the measurement of bent posture is mostly performed in the static state using only photography or radiography [10,11,12]. These modalities cannot accurately measure the flexion angle during gait, which limits the evaluation of treatment effects.

Sèze et al. [13] reported the results of lateral posture measurement during gait by using a three-dimensional (3D) camera system, an optoelectronic system for kinematic analysis, in patients with PD with camptocormia. They found that the horizontal distance between the C7 and S1 bones, which indicates the degree of postural bending, was significantly increased in the late phase compared with the early phase of gait. They also suggested that postural measurements obtained during walking could more accurately reveal the deleterious effects of camptocormia on the quality of life than measurements taken in the static state. However, such a measurement system is expensive and can only be used in specific environments, making it difficult to utilize in daily clinical practice.

Wearable sensors have recently been used for various purposes in the medical field. There have been attempts to quantify postural stabilities using wearable sensors in patients with PD [14,15]. Furthermore, there have been other attempts to use wearable sensors for home-based gait training in patients with PD [16,17]. Our previous study showed that measurement of the forward flexion angles of the neck and back using wearable sensors is a valid method compared with the use of a 3D camera system during simulated parkinsonian gait in healthy volunteers [18].

It is known that various cueing modalities have a positive effect on exercise training in patients with PD [19,20]. Verbal, visual or sensory cues are sometimes helpful to correct a patient’s posture during walking. There have been many attempts of cueing to improve the gait and posture of patients with PD including open-loop cueing and further closed-loop cueing that provides feedback in a real-time manner [21]. However, continuous feedback on posture is difficult to provide during activities. Wearable sensors not only can be utilized for quantitatively evaluating characteristic postural abnormalities in patients with PD during gait, but also can be used for the purpose of training to maintain proper posture during walking by providing vibratory sensory stimulation through sensors. Since the sensors that we validated in our previous study can accurately measure the sagittal angle in real-time even during gait, it is expected that the sensors can provide continuous feedback on posture in patients with PD. Therefore, the objectives of this preliminary study were to measure the degree of stooped posture by using wearable sensors during walking in patients with parkinsonism and to investigate whether sensory feedback using vibration from sensor modulescan improve the posture of these patients.

The paper is structured as follows. Section 1 describes a brief introduction related to the stooped posture in patients with PD and the need for wearable sensors that can provide feedback in a real-time manner. Materials and methods including study subjects, design, how the sensor works, outcome measures and method of statistical analysis are presented in Section 2. Then, the results of this study are depicted in Section 3. Finally, in Section 4 and Section 5, we will discuss the results obtained through the study and present the conclusions of this study.

## 2. Materials and Methods

### 2.1. Participants

Patients with parkinsonism with stooped posture were recruited from the rehabilitation outpatient clinic of our hospital. The inclusion criteria were (1) clinically diagnosed parkinsonism including idiopathic PD, multiple system atrophy (MSA) and progressive bulbar palsy; (2) stooped posture due to forward flexion of the thoracolumbar region; (3) ability to walk independently; and (4) age > 18 years. The diagnostic criteria for idiopathic PD followed UK Parkinson’s Disease Society Brain Bank criteria [22] and the diagnosis for MSA followed 2nd consensus of the Gilman criteria [23]. Stooped posture was defined as a stage 2 (Definite flexion, scoliosis, or leaning to one side, but patient can correct posture to normal posture when asked to do so) or higher in the “3.13 posture” item on Movement Disorder Society–Unified Parkinsons Disease Rating Scale (MDS-UPDRS) part III [24]. The exclusion criteria were (1) fixed kyphosis that cannot be improved by leaning or lying down; (2) difficulty in measuring posture because of severe dyskinesia or tremors; (3) cognitive impairment that impedes understanding the research contents; and (4) other neurological, orthopedic, or medical disease that restricts gait. Before the study, the patients’ characteristics, diagnosis, disease duration and HnY stage were collected. All patients provided written informed consent and voluntarily agreed to participate in the study. The study protocol was approved by our institutional review board (approval No. 1706-002-855).

### 2.2. Study Design

**Figure 1:**
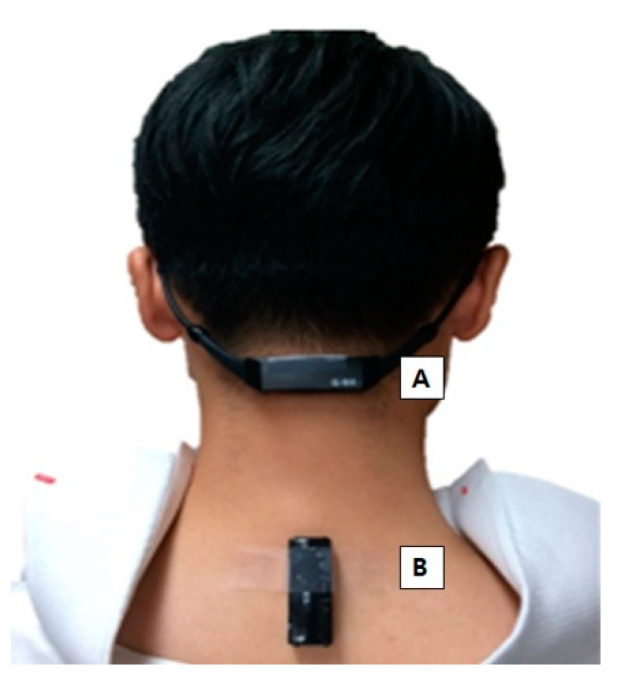
(**A**) Neck sensor on the C2-3 spinous process area. (**B**) Back sensor on the C7-T1 spinous process area, just below the prominence of C7.

The present study was designed as a preliminary investigation of the feasibility and efficacy of wearable sensors for the assessment of stooped posture in patients with parkinsonism. First, two wearable sensors were attached, one at the C2-3 spinous process area (neck sensor) and the other in the C7-T1 spinous process area (back sensor) of the patients (Figure 1). Then, each patient underwent a 6 min walk test two times (control session, vibration session). The sequence of sessions was randomized according to the order of participation and there was a 10 min break between the sessions. The absolute sagittal angles in the most upright posture before walking and the mean changes of the sagittal angles during walking were measured in each patient. For safety, all patients wore a waist belt with a handle and a researcher accompanied each patient while walking.

In the control session, the wearable sensors only measured the flexion angle without providing sensory feedback. In the vibration session, the sensors not only measured the flexion angle but also stimulated the patients with vibration. Sensory feedback was provided through vibrations from the neck sensor when the sagittal angle of the neck became lower than the threshold angle. The threshold angle was set to 10° or 20° according to the judgment of a physiatrist considering the patient’s performance. The back sensor measured the flexion angle but did not provide any vibration. Before beginning the session, the patients were instructed to correct their posture according to the sensory cue from the neck sensor.

### 2.3. Measurement of Angles and Vibration Feedback

The sensor module includes a three-axis accelerometer (BMA250; Bosch, Blaichach, Germany), a Bluetooth integrated microprocessor, a flash memory and a vibrator. The accelerometer has a programmable functionality ranging from ±2 g to ±16 g with 0.8 mg/√Hz output noise. In this application, the sensor was configured to work at ±2 g range with a sensitivity of 256 LSB/g. The flexion angle can be estimated by observing the projections of the gravitational acceleration on the sensor’s axes. The estimated flexion angle was stored in the flash memory each second then the data was analyzed offline using MATLAB. Before walking, the patients were asked to stand in the most upright posture. The absolute sagittal angles were measured as the baseline neck and back angles (90° when standing perpendicular to the ground).

The sensors were calibrated in the most upright posture before the patients started walking (defined as 0°). Then, the sensors continuously recorded the sagittal angles at 20 Hz and averaged the data at every second during a 6 min walk test. To eliminate artifacts from walking and tremor motions in patients with parkinsonism, an averaging algorithm was applied to the accelerometers.

It was proved that the human walking movement is sinusoidal in the lateral and vertical direction where the vertical movement frequency is double the lateral movement frequency [25]. That means the movement artifacts in each axis can be easily eliminated from the accelerometer data by averaging the data in each gait cycle. Since the forward acceleration in 6 min walking test is approximated to be linear, the accelerometer data after human artifact removal only represents the gravitational force. Thus, the inclination angle α can be estimated from the accelerometer output $a={\left[a_{x}\;a_{y}\;a_{z}\right]}^{T}\in {ℜ}^{3}$ by the following equation

$$\alpha =\tan^{-1}\frac{{\bar{a}}_{x}}{\sqrt{{\bar{a}}_{y}^{2}+{\bar{a}}_{z}^{2}}}$$

where ${\bar{a}}_{i}=\frac{1}{N}\overset{N}{\underset{k=1}{\sum }}a_{i_{k}}$ is the average of N accelerometer data in i -axis (i={x,y,z}). In accordance with the results concluded in the previous studies [26,27], the average walking cadence of the Parkinson’s disease patients is about 2 steps per second (103~129 steps per minute). In other words, one gait cycle is approximately one second. Therefore, N was chosen as 20 to eliminate the movement artifacts in each sensor axis since the sensor samples at 20 Hz. This measurement has been validated in healthy participants by comparing it with a 3D camera system with markers (Optitrack), with mean absolute errors of 0.9–1.5° [18].

In the vibration session, sensory cue in the form of vibration feedback was provided from the neck sensor based on real-time angle determination. The vibration was provided as soon as the neck angle exceeded the threshold in each patient. The vibration signal had a 140 Hz frequency, 200 ms duration and 0.012 W power. The signal was continuously provided at 1 s intervals until the patient corrects the posture to below the threshold.

### 2.4. Outcome Measures

The changes of neck and back flexion angles were recorded every second during the control and vibration sessions. Individual data was graphed to examine the feasibility and efficacy of the sensors in each patient. The average changes of forward flexion angle of the neck (Mean_N) and back (Mean_B) were calculated from the data. The neck and back flexion angles in the most upright posture (Base_N, Base_B) before beginning the sessions and the total distance in the 6 min walk test were recorded. Heart rate (HR) and ratings of perceived exertion (RPE) in the Borg scale were also measured at the beginning and end of each test.

### 2.5. Statistical Analysis

The normality of data was evaluated using the Shapiro–Wilk test to determine whether the distribution of values (Base_N, Base_B, Mean_N, Mean_B, HR, RPE and distance) followed a normal distribution. Paired *t*-test and Wilcoxon signed-rank test were used to compare the values between the vibration and control sessions according to the normality of each value. To measure the effect size, Cohen-d or Cliff-delta index was used according to the normality of each value. Statistical analysis was performed using SPSS version 19 (SPSS Inc., Chicago, IL, USA) and *p* < 0.05 was considered statistically significant.

## 3. Results

### 3.1. Participants’ Characteristics

A total of 12 patients who met the criteria participated in the study. Two patients were excluded from the analysis because they made unintended thoracolumbar flexion and extension movements during the test. Finally, the data of 10 patients (two male patients, eight female patients) were collected. The mean patient age was 70.6 ± 5.76 years. Of the 10 patients, nine had idiopathic PD and one had MSA-parkinsonian type. The median HnY stage was 2.75 (interquartile range 0.5) and the mean duration of disease was 7.16 ± 4.35 years. The patients’ demographics and details of diagnosis are summarized in Table 1.

### 3.2. Measurements of Flexion Angles

The results of the study are shown in Table 2. Four patients (#1, #2, #5, #6) began with the control session and six patients (#3, #4, #7, #8, #9, #10) started with the vibration session. The threshold of vibration was 20° in patients #1–#4 and 10° in patients #5–#10. Base_N and Base_B in the vibration session (96.5 ± 5.25° and 73.0 ± 17.84°) were both significantly higher than those in the control session (90.7 ± 8.84° and 70.0 ± 15.15°) (*p* = 0.026 and 0.043, respectively).

The neck flexion and back flexion were somewhat aggravated during gait, although the average change was <10° in most patients. Only two patients (#7, #10) showed a >10° change in the neck and back angles in both sessions. The number of vibrations was >200 in two patients (#7, #10) and 10–30 in five patients (#1, #2, #6, #8, #9), whereas there was no vibration stimulation in three patients (#3, #4, #5).

Mean_N in the control session was 8.32 ± 7.84° and that in the vibration session was 6.88 ± 7.55°. On the other hand, Mean_B in the control session was 6.65 ± 6.63° and that in the vibration session was 8.75 ± 11.93°. As both Mean_N and Mean_B data followed a normal distribution, paired *t*-test and Cohen-d were used. Both results were not statistically significant (*p* = 0.449 and 0.319, respectively) and the effect sizes were 0.250 and 0.334 respectively.

The mean distance in the 6 min walk test was almost the same in the two sessions (255 m in the control session, 254 m in the vibration session). The mean change of HR was −1.8 in the control session and 2.5 in the vibration session, which showed a statistically significant difference (*p* = 0.038). The mean change of RPE was 4.2 and 2.5 in the control and vibration sessions, respectively, with no statistically significant difference (*p* = 0.218).

### 3.3. Response to Sensory Feedback in Each Case

Three patients (#3, #4, #5) had no sensory feedback because the neck flexion angle during the vibration session never exceeded the threshold. Two patients (#7, #10) did not respond to the stimulation although they received >200 vibrations. In addition, three patients (#2, #8, #9) sometimes immediately responded to the vibration but occasionally responded after several vibratory stimuli. However, two patients (#1, #6) showed immediate response to the sensory feedback.

**Figure 2:**
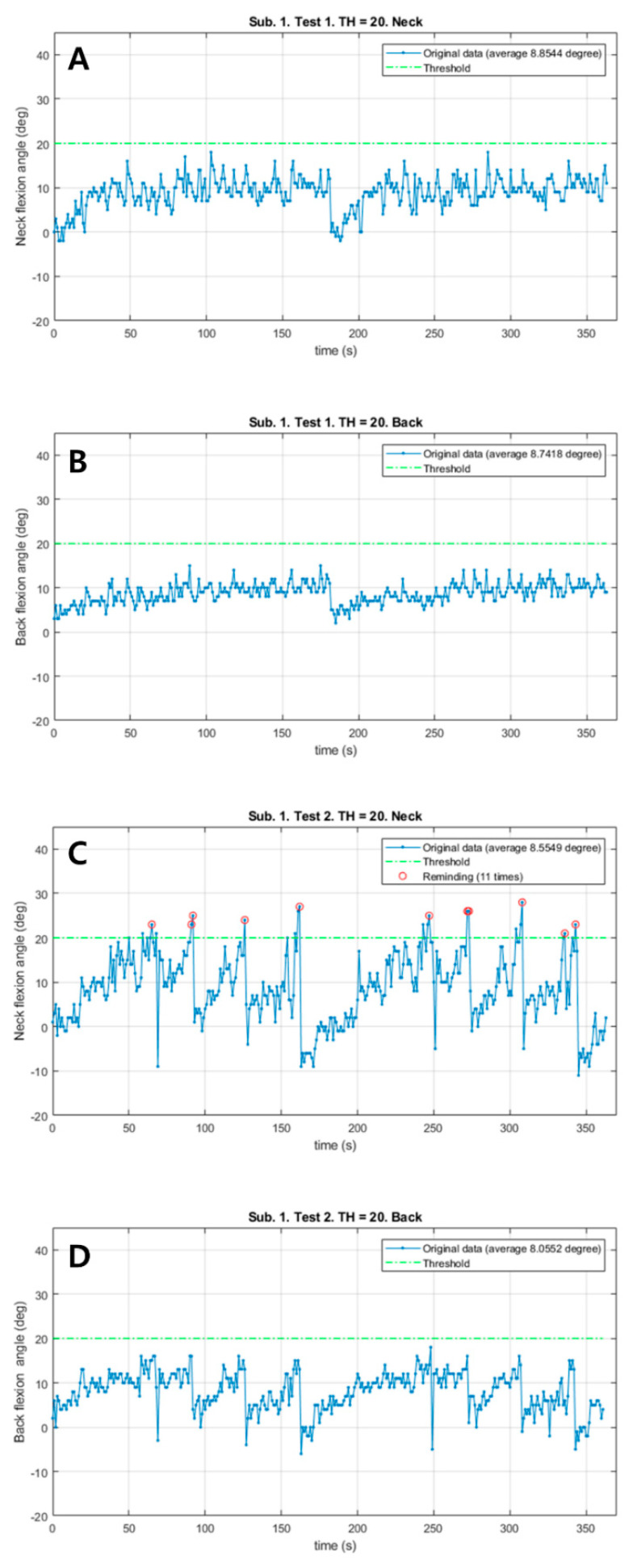
The changes of neck and back flexion angles continuously measured during 6 min walk test in patient #1. (**A**) The changes of neck flexion angles in the control session. (**B**) The changes of back flexion angles in the control session. (**C**) The changes of neck flexion angles in the vibration session. (**D**) The changes of back flexion angles in the vibration session. Red circles: Reminding the patient by vibration feedback.

Patient #1 responded immediately to the vibrations from the neck sensor and corrected both the neck and back flexion postures in the vibration session with a total of 11 vibrations (Figure 2). However, the neck flexion angle exceeded the 20° threshold only in the vibration session. Both Mean_N and Mean_B during walking slightly decreased in the vibration session (0.29° in the neck, 0.68° in the back). There were no remarkable differences in the total walk distance, HR and RPE.

**Figure 3:**
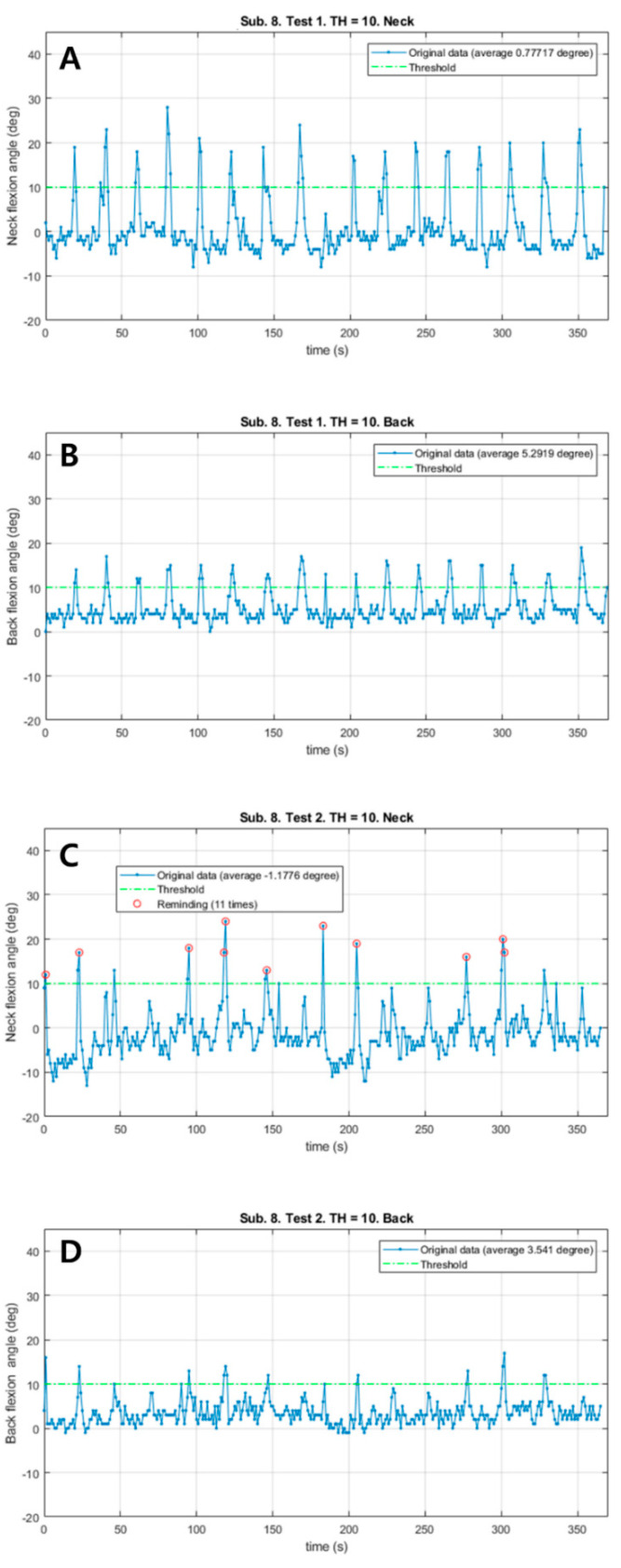
The changes of neck and back flexion angles continuously measured during 6 min walk test in patient #6. (**A**) The changes of neck flexion angles in the control session. (**B**) The changes of back flexion angles in the control session. (**C**) The changes of neck flexion angles in the vibration session. (**D**) The changes of back flexion angles in the vibration session. Red circles: Reminding the patient by vibration feedback.

Patient #6 also responded immediately to a total of 11 vibrations and corrected the postures (Figure 3). The neck flexion angle exceeded the threshold of 10° in both sessions. The patient corrected the posture much sooner in the vibration session than in the control session. In the vibration session, Mean_N and Mean_B decreased (1.96° in the neck, 1.75° in the back). There were no remarkable differences in HR and RPE; however, the distance in the vibration session was 26 m shorter.

## 4. Discussion

This study proved that wearable sensors can effectively measure posture during walking in real time. The patients underwent two test sessions, a control session without vibration stimulation and a vibration session with vibration stimulation and the results were compared to evaluate whether the patients could respond to vibration stimuli and correct their stooped posture. The neck flexion and back flexion were slightly aggravated during gait, but the average change was <10° in most patients in both measurement sessions. Therefore, it was difficult to evaluate the effect of sensory feedback through vibration. However, some patients showed immediate response to the feedback and corrected their posture during gait.

The cause of stooped posture in PD is still unclear and various treatments have been proposed to correct this abnormality. Rehabilitation therapy such as posture correction training is known to be somewhat helpful [10,28]. Conventional methods such as the use of a low-slung backpack or a cruciform anterior spinal hyperextension brace have also been applied [29,30]. Recently, it was reported that levodopa was effective in alleviating the degree of stooped posture in patients with PD [10]. In addition, magnetic stimulation of the spine [11], high-frequency deep-brain stimulation [31], botulinum toxin injection [32] and orthopedic surgical correction [33] have been proposed for the treatment of camptocormia.

We used vibration feedback just for alarming because sensory feedback can improve the posture of patients as previous studies have shown [21]. However, it has been suggested that vibration itself may improve posture and gait in patients with PD. Serio et al. [34] found that focal vibration training associated with a rehabilitative protocol can reduce the frequency of falls in patients with PD. The authors suggest that vibrations can influence proprioceptive sensation, motor control and standing posture. De Nunzio et al. [35] have reported that vibration on the erector spinae increased the stride length suggesting that vibration was a proprioceptive cue. In addition, it is known that tendon vibration can alter proprioceptive estimates of position [36]. Although we used vibration stimulation just for alarming that the flexion angle exceeded the threshold, the vibration stimulation itself may affect the patient’s posture.

The differences of the average neck and back flexion angles between the control session and the vibration session, which is the primary outcome of this study, were not statistically significant. As three of the 10 patients had no opportunity to correct their posture in response to vibratory stimulation, it would be more meaningful to analyze the data of individual patients than to compare the mean values of all patients. The baseline neck and back flexion angles in the vibration session were significantly higher than those in the control session. Although this result may not be directly related to vibration feedback, it should be noted that patients may have been more nervous or trying to extend their posture regardless of sensory feedback in the vibration session.

Patients #1 and #6 immediately responded to vibration stimulation and corrected their posture. In particular, as the neck flexion angle in patient #6 exceeded the threshold in both the control and vibration sessions, this represents a good case for comparing the responses to the sensory cue in a state in which similar postural changes occurred. Although the patient sometimes corrected the flexion angle when the neck threshold was exceeded in the control session, the flexion angle was adjusted much more quickly when the threshold was exceeded in the vibration session (Figure 3). Patients #2, #8 and #9 sometimes, but not always, corrected their posture by immediately responding to vibration stimulation. Patients #3, #4 and #5 never reached a flexion angle of >10° during the test; therefore, it was difficult to evaluate the adequacy of the sensory cue. Patients #7 and #10 did not correct their posture as a response to sustained stimulation. Patient #7 was the only patient diagnosed with MSA. Recently, there have been studies showing that patients with MSA have a higher likelihood of having depression and lower executive function than patients with idiopathic PD [37,38]. This may be one of the reasons why patient #7 did not respond to vibration feedback.

Ginis et al. [16]. analyzed the patients’ gait pattern by using an inertial measurement unit for home-based training and provided verbal cues or visual cues to the patients through a smartphone app. They demonstrated that the balance function was better and the improvement in quality of life was higher in the experimental group than in the control group. In addition, Espay et al. [17] used a virtual reality cueing device with a multiaxial accelerometer for home use to assess the patients’ walking pattern and provided visual and auditory cues via the goggles and earphones based on the data from the multiaxial accelerometer. They demonstrated that this method can improve the patients’ gait pattern. Wegen et al. [39] performed a pilot study in 15 patients and applied a wearable sensor for home-based training aiming to correct the flexion posture of the patients by providing vibration feedback if the flexion angle exceeded a certain angle. They observed a statistically significant improvement of flexion angle in the vibration session compared with the control session. The differences of their study from ours were as follows: (1) the control session was conducted in the first 1 week as a home-based training and the vibration session was conducted in the following 1 week; (2) one sensor was attached to the sternum; and (3) the vibration threshold was set individually. The reasons for the significant improvement in flexion angle might be the long-term observations at home, the possible learning effect from the non-randomized order of sessions and the individualized vibration threshold determined by the therapist (subjective judgment).

This study has some limitations. First, the number of study subjects was small. As a preliminary study, we mainly focused on the feasibility of wearable sensors for the assessment of stooped posture in patients with parkinsonism and the response to sensory feedback in each case. Second, it is possible that the stimulus intensity was too weak for the patient to recognize the vibration. In a recent study, it was suggested that sensory perception is impaired in patients with PD compared with healthy people [40]. In this study, the strength of the vibration stimulus was sufficient to be recognized by healthy adults; however, it is unclear whether the strength of vibration was sufficient for patients with PD. In future studies, it will be important to evaluate the intensity of vibration stimulation in patients with PD and to confirm that the patients can recognize the vibration well. Third, considering that patients had HnY stage 2 to 3 parkinsonism, a 10 min rest time after the first session may not have been sufficient. Particularly, this study did not merely involve walking. As the aim of this study was to evaluate posture during walking, the patients needed to focus on responding to vibratory stimuli and correct their posture. Finally, it was difficult to assess the effect of sensory feedback because the patients generally did not exhibit severe postural abnormalities, as we expected. This was probably because the patients were more nervous and exerting more effort than usual to keep the extended posture. Furthermore, the 6 min walk test might not have been long enough. Therefore, further studies will be required to evaluate the effectiveness of these sensors in daily life.

## 5. Conclusions

This preliminary study suggests that stooped posture could be quantitatively measured during gait by using wearable sensors in patients with parkinsonism. From the review of previous literature, it is clear that various cueing strategies improve posture in patients with PD. In order to overcome the limitations of open-loop cueing, which is provided irrespective of patient’s performance, closed-loop cueing, which provides real-time feedback according to the patient’s situation, is being actively researched. As part of that paradigm, we developed effective wearable sensors that can evaluate the patient’s posture and give immediate feedback in everyday life. Although the results were not statistically significant in this study, sensory feedback through vibration from sensor modules may be helpful in correcting posture during gait in selected patients. Further studies are necessary to confirm the effect of sensory feedback by these sensors on stooped posture in patients with parkinsonism.

## Figures and Tables

**Table 1:** Demographics and diagnosis.

Patient	Sex	Age	BMI	Diagnosis	HnY	Duration
#1	F	74	23.1	IPD	2.5	2.9 years
#2	F	72	22.8	IPD	3	1.8 years
#3	F	74	16.4	IPD	2.5	11.1 years
#4	F	79	19.4	IPD	3	3.1 years
#5	F	65	17.0	IPD	2	12.1 years
#6	F	72	22.0	IPD	3	11.1 years
#7	M	66	29.1	MSA-P	3	4.0 years
#8	F	60	22.7	IPD	2.5	11.0 years
#9	F	76	28.0	IPD	3	3.6 years
#10	M	68	22.2	IPD	2.5	10.9 years

IPD, Idiopathic Parkinson’s Disease; MSA-P, multiple system atrophy–parkinsonian type; HnY, Hoehn and Yahr scale.

**Table 2:** Individual data of study participants.

Patient	Control					Vibration						
	Base_N	Base_B	Mean_N	Mean_B	Distance	Base_N	Base_B	Mean_N	Mean_B	Distance	Vib #	Thr
#1	87	72	8.85	8.74	219	101	75	8.56	8.06	218	11	20
#2	100	63	3.2	2.52	208	105	62	13.15	5.57	268	17	20
#3	104	55	−0.33	5.87	341	102	52	6.75	5.67	360	0	20
#4	88	53	9.57	6.54	251	92	55	3.53	8.69	216	0	20
#5	97	60	2.85	2.55	340	97	62	−2.6	−1.68	330	0	10
#6	99	52	0.78	5.29	263	99	55	−1.18	3.54	226	11	10
#7	88	83	20.21	16.46	116	91	89	12.99	23.11	133	240	10
#8	77	89	9.72	−2.75	276	96	91	5.39	1.03	289	27	10
#9	86	87	5.45	2.29	246	89	91	0.39	−2.37	233	24	10
#10	81	86	22.87	18.94	290	93	98	21.82	35.88	267	290	10
Mean	90.7	70.0	8.32	6.65	255	96.5	73.0	6.88	8.75	254	62	
SD	8.84	15.15	7.84	6.63	66.06	5.25	17.84	7.55	11.93	64.30	108.06	

Base_N, baseline neck flexion angle in upright posture; Base_B, baseline back flexion angle in upright posture; Mean_N, mean change of neck flexion angle during gait; Mean_B, mean change of back flexion angle during gait; Vib #, the number of vibration; Thr, threshold; HR, end of test HR–beginning of test HR; RPE, end of test RPE–beginning of test R.

## Data Availability

The data presented in this study are available on request from the corresponding author. The data are not publicly available due to privacy.
